# Interplay Between the N-Terminal Domains of *Arabidopsis* Starch Synthase 3 Determines the Interaction of the Enzyme With the Starch Granule

**DOI:** 10.3389/fpls.2021.704161

**Published:** 2021-09-23

**Authors:** Francisco M. Gámez-Arjona, Ángel Mérida

**Affiliations:** Institute of Plant Biochemistry and Photosynthesis, Consejo Superior de Investigaciones Científicas-University of Sevilla, Sevilla, Spain

**Keywords:** starch, starch binding domain, starch synthase 3, *Arabidopsis*, starch metabolism

## Abstract

The elongation of the linear chains of starch is undertaken by starch synthases. class 3 of starch synthase (SS3) has a specific feature: a long N-terminal region containing starch binding domains (SBDs). In this work, we analyze *in vivo* the contribution of these domains to the localization pattern of the enzyme. For this purpose, we divided the N-terminal region of *Arabidopsis* SS3 in three domains: D1, D2, and D3 (each of which contains an SBD and a coiled-coil site). Our analyses indicate that the N-terminal region is sufficient to determine the same localization pattern observed with the full-length protein. D2 binds tightly the polypeptide to the polymer and it is necessary the contribution of D1 and D3 to avoid the polypeptide to be trapped in the growing polymer. The localization pattern of Arabidopsis SS3 appears to be the result of the counterbalanced action of the different domains present in its N-terminal region.

## Introduction

Most plants store any excess energy and carbon produced by photosynthesis in the form of starch. Starch granules are semicrystalline structures composed of two different α-glucan polymers: amylose and amylopectin. Both are composed of linear chains of α-1,4-linked glucose units branched through α-1,6 bonds (Buléon et al., 1998). Amylose, the minor component, has an amorphous structure and few branches, while the major partner, amylopectin, is moderately branched and gives rise to the semicrystalline structure of the starch granule (Buléon et al., 1998). The synthesis of starch requires the concerted action of different activities: the elongation of the chains of the glucan polymer by starch synthases (SSs, ADP-Glc: α-1,4 glucan α-4-glucosyltransferases, EC 2.4.1.21), the synthesis of branches by starch branching enzyme (SBE, α-1,4 glucan branching enzyme, EC 2.4.1.18) and the removal of excess branches (to allow the crystallization of amylopectin branches grouped in clusters) by starch debranching enzyme (DBE or Isoamylase, EC 3.2.1.68). All these enzymes have different isoforms. Thus, *Arabidopsis* contains six classes of SSs, GBSS (granule-bound starch synthase), SS1, SS2, SS3, SS4, and SS5. In some cases, such as cereals, maize or rice, there are more than one isoform for each class (Jeon et al., 2010). GBSS is responsible for the synthesis of amylose (Kuipers et al., 1994; Denyer et al., 1995; Nakamura et al., 1995), SS4 and SS5 are involved in the process of initiation of the starch granule (Roldán et al., 2007; Abt et al., 2020), and the remaining SSs elongate the different chains of the amylopectin molecule (Szydlowski et al., 2011).

SS3 was first identified in potato (Edwards et al., 1995; Abel et al., 1996) and is present in all starch-accumulating organisms (Ball and Morell, 2003). The suppression of SS3 activity in *Chlamydomonas* (Fontaine et al., 1993) and potato (Edwards et al., 1999) has a major impact on the synthesis of amylopectin, resulting in a polymer with a modified chain-length distribution and the reduced synthesis of starch. *Arabidopsis* mutants lacking SS3 (AtSS3 mutants) activity showed significant increases in the frequency of linear chains of amylopectin with a degree of polymerization >60. Furthermore, the total SS activity is increased in *Atss3* mutants, with plants showing greater starch content in the leaves during the light period, suggesting that SS3 has a negative regulatory function on the biosynthesis of transitory starch in *Arabidopsis* (Zhang et al., 2005). Our group has also shown that SS3 can partially substitute the function of SS4 in the initiation of the starch granule, and therefore most of the chloroplasts of the *ss3-ss4* double mutant are devoid of starch (Szydlowski et al., 2009). Other authors report that SS3, together with other enzymes involved in starch metabolism (SS2, SBE2a and SBE2b), form a multiprotein complex in maize amyloplasts, indicating that they may modulate each other's activities (Hennen-Bierwagen et al., 2009).

SS3 contains different functional domains that enable it to perform the functions described above. Like other SSs, SS3 is a GT-B-fold glycosyltransferase, classified within family GT5 in the CAZy database (Coutinho et al., 2003). The C-terminal region is homologous to other SSs and contains the catalytic (GT5) and the glycosyltransferase (GT1) domains. SS3, however, has a long N-terminal region, exclusive to its class with three Starch Binding Domains (SBDs) belonging to the family 53 of Carbohydrate Binding Modules (CBM) (Palopoli et al., 2006) (http://www.cazy.org). These SBDs have been comprehensively analyzed *in vitro* both in *Arabidopsis* (Valdez et al., 2008; Wayllace et al., 2010) and the picoalgae *Ostreococcus tauri* (Barchiesi et al., 2015, 2018). These studies indicate that they modulate the catalytic activity of the C-terminal region, and highlight the importance of SBDs 2 and 3 in binding to starch and in the regulation and catalysis of SS3 (Wayllace et al., 2010). Computational analysis of the N-terminal region of maize SS3 has identified two coiled-coil (CC) domains, which typically function to mediate protein-protein interactions (Rose and Meier, 2004). The two CC domains flank the central SBD, which Wayllace et al. (2010) indicate to be the most significant domain with respect to substrate affinity. These analyses suggest that the N-terminal region of SS3 may contain regions that undertake protein-protein interactions and non-catalytic glucan binding (Hennen-Bierwagen et al., 2009).

We have previously shown that AtSS3 surrounds the surface of the starch granule and, after disrupting the chloroplasts, it is found in the soluble, stromal fraction; it is not detected in the membrane or starch fractions (Gámez-Arjona et al., 2014). The present work shows that the N-terminal region of AtSS3 is responsible for the localization pattern of the enzyme. This region contains three predicted CC domains and three SBDs. We show that the region containing the CC and SBD 1 and 2 are necessary, and sufficient, for correct localization (AtSS3 localization) to be achieved. Those containing the CC and SBD 2 and 3, in the absence of CC1 and SBD1, change the localization pattern, with the polypeptide appearing tightly bound to the starch granule.

## Materials and Methods

### Plant Materials and Growth Conditions

*Nicotiana benthamiana* plants were grown in soil (in pots) in a greenhouse under a 16-h light/8-h dark cycle (light intensity 250 μmoles m^−2^s^−1^) at 22°C.

### Plasmids Construction

cDNAs required for the transient expression of truncated versions of AtSS3 in *N. benthamiana* were cloned using the Gateway system (ThermoFisher Scientific). The different fragments were amplified using the oligonucleotides shown in Supplementary Table 1. The sequenced fragments were cloned into the pDONR207 plasmid (ThermoFisher Scientific) *via* BP clonase reaction and subsequently transferred to pEarleyGate103 (Earley et al., 2006) *via* a LR clonase reaction to allow the C-terminus translational fusion of the fragments to GFP. An overlapping PCR strategy was employed to clone fragments D13, D23, D2, and D3 and to fuse the AtSS3 chloroplast transit peptide (CTP) with the sequence of interest.

### Transient Expression in *N. benthamiana*

Corresponding transgene vectors were electroporated into *Agrobacterium tumefaciens* strain C58 (Wood et al., 2001). The agroinfiltration of *N. benthamiana* leaves was assessed as previously described (Gámez-Arjona et al., 2014).

### Confocal Microscopy

A DM6000 confocal laser-scanning microscope (Leica Microsystems) equipped with a ×63 oil immersion objective and a ×20 objective was used to detect the localization of the GFP fusion proteins. GFP and chlorophyll autofluorescence imaging was performed by exciting the cells with an argon laser at 488 nm and detecting fluorescence emissions at 500–525 nm and 630–690 nm, respectively.

### AtSS3 Fragments Localization Analysis by Immunoblotting

The fragments D123, D12, D2, and D23, cloned into pEarlyGate 103 vector, were transiently expressed in *N. benthamiana* leaves by agroinfiltration as described above. Leaf material (500 mg) was harvested 2 days after infiltration at the same time of the day/night cycle to avoid differences of starch accumulation between samples analyzed. Samples were disrupted in liquid nitrogen with a mortar and pestle. Extracts were resuspended in 500 μl PD buffer (20 mM HEPES-KOH, pH 7.6, 80 mM NaCl, 1 mM MgCl_2_, and 1 mM DTT) supplemented with 2 μl protease inhibitor cocktail (SIGMA) and filtered through Miracloth (Merck). The extract was centrifuged at 12,000 × g at 4°C for 20 min over 1 ml Percoll 90% diluted in PD buffer. The pellet (starch granules) was washed with 500 μl PD buffer supplemented with 0.2% Triton X-100 and pelleted again by centrifugation at 12,000 × g at 4°C for 20 min. The pellet was resuspended in 20 μl preloading buffer (50 mM Tris-HCl, pH 6.8, 2% SDS) and boiled for 10 min. Samples were then centrifuged a 12,000 × g at 4°C for 10 min to eliminate debris. Protein in the supernatant fractions was determined according to the Lowry method (Lowry et al., 1951) and 2 μg of each sample were analyzed by SDS-PAGE (Laemmli, 1970) (15% total acrylamide) in a Mini-Protean Tetra cell (BIO-RAD), transferred to nitrocellulose membrane by electroblotting in a Trans-Blot Turbo transfer cell (BIO-RAD) and immunoblotted using anti-His5 horseradish peroxidase conjugate antibody (QIAGEN) at 1:2,000 dilution. The chemiluminescence was visualized using a Chemidoc Imaging System (BIO-RAD) running Quantity One software (BIO-RAD).

## Results

### The N-Terminal Region of SS3 Determines the Localization of the Enzyme

**Figure 1:**
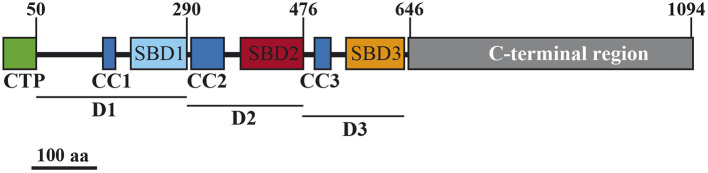
Schematic representation of the AtSS3 protein. Numbers at the top indicate amino acid positions. SBD, starch-binding domain; CC, coiled-coils sites; CTP, chloroplast transit peptide. D1, D2, and D3 are the domains employed in this study.

AtSS3 has two well-differentiated regions, the C-terminal region, containing the catalytic and substrate binding sites, and the N-terminal region, which contains three SBDs (SBD1 from amino acid 205 to 290, SBD2 from 380 to 476, and SBD3 from 547 to 637, according to TAIR – http://www.arabidopsis.org-) plus CC domains of imprecise localization. The DeepCoil tool of the web service for protein bioinformatic analysis MPI Bioinformatics Toolkit (https://toolkit.tuebingen.mpg.de) (Zimmermann et al., 2018) was used to predict the position of these CC. Three were predicted: CC1 from amino acid 161 to 177, CC2 from 301 to 349, and CC3 from 493 to 517. Figure 1 shows the different regions and domains of AtSS3.

**Figure 2:**
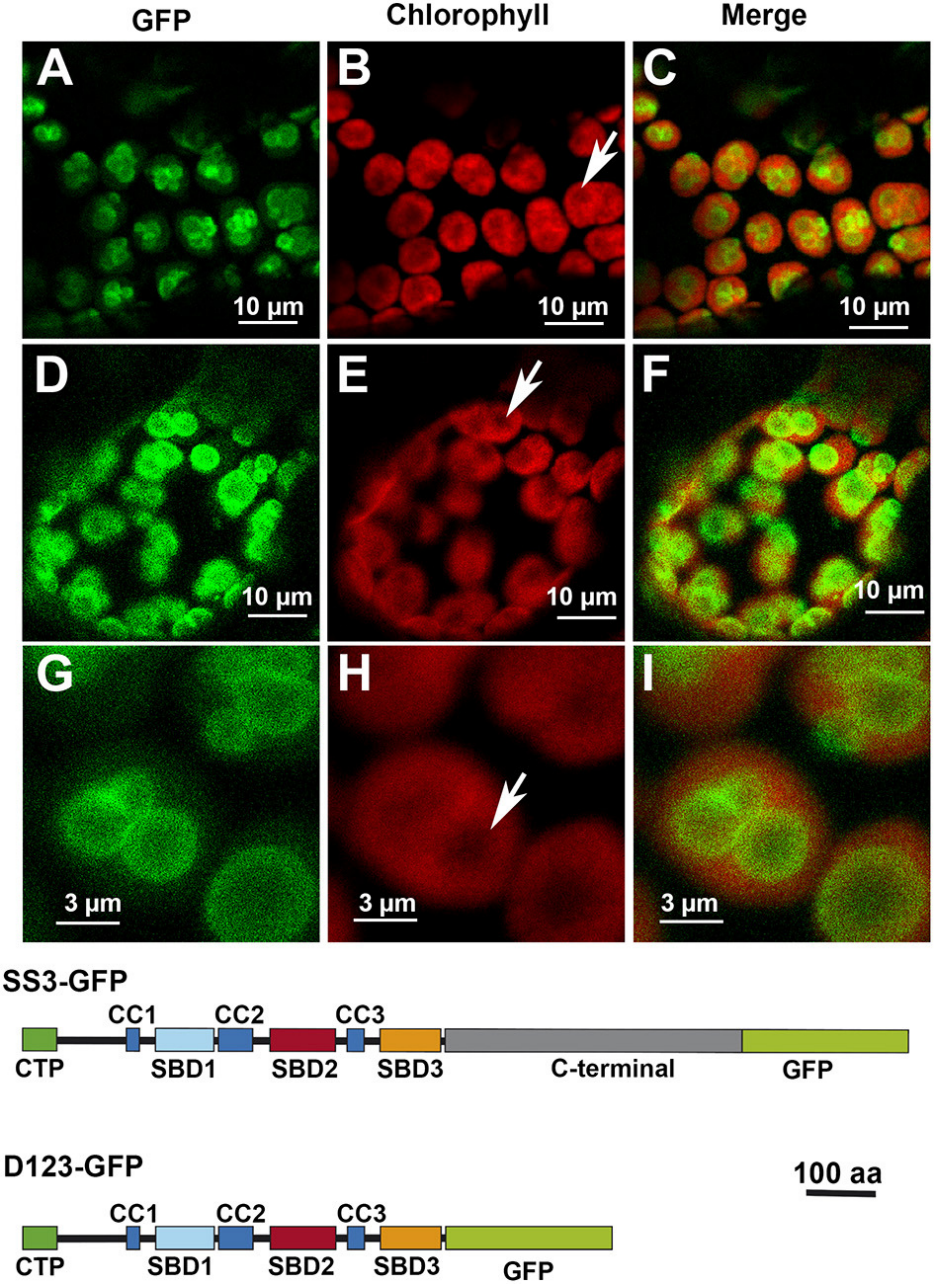
Localization of full-length AtSS3 **(A–C)** and the N-terminal region of AtSS3 **(D–I)** fused to GFP in *Nicotiana benthamiana* chloroplasts. GFP column: fluorescence of the GFP marker. Chlorophyll column: autofluorescence of the chlorophyll. Merge column: merged images of previous columns. Bottom: structures of the constructs fused to GFP. SS3-GFP: full-length AtSS3 fused to GFP. D123-GFP, N-terminal region of AtSS3 fused to GFP. The white arrows indicate the presence of a starch granule.

We have previously shown that each *Arabidopsis* SS displays a specific pattern of localization in the chloroplast. The localization of AtSS3 has been indicated as restricted to the region surrounding the starch granules in the chloroplast (Gámez-Arjona et al., 2014). Using the same technical approach as reported in this latter paper, analyses were made to determine whether the N-terminal region of AtSS3 is sufficient to determine the localization pattern observed for the full-length protein. Figure 2 shows that the N-terminal region of AtSS3 (denoted D123) displayed the same localization pattern than the complete protein. Figures 2G–I show magnified images of the localization of D123-GFP, with the polypeptide surrounding the starch granule. Starch granules appear as black areas lacking chlorophyll autofluorescence (Figure 2H).

### Contribution of Each Domain to the Localization Pattern of AtSS3

To analyze the contribution of the different domains in the AtSS3 N-terminal region to the localization pattern of the full protein, the region was divided into three domains: D1, containing CC1 and SBD1, D2, containing CC2 and SBD2, and D3, containing CC3 and SBD3 (see Figure 1). The effect of eliminating one or two domains on the pattern of localization was then tested.

**Figure 3:**
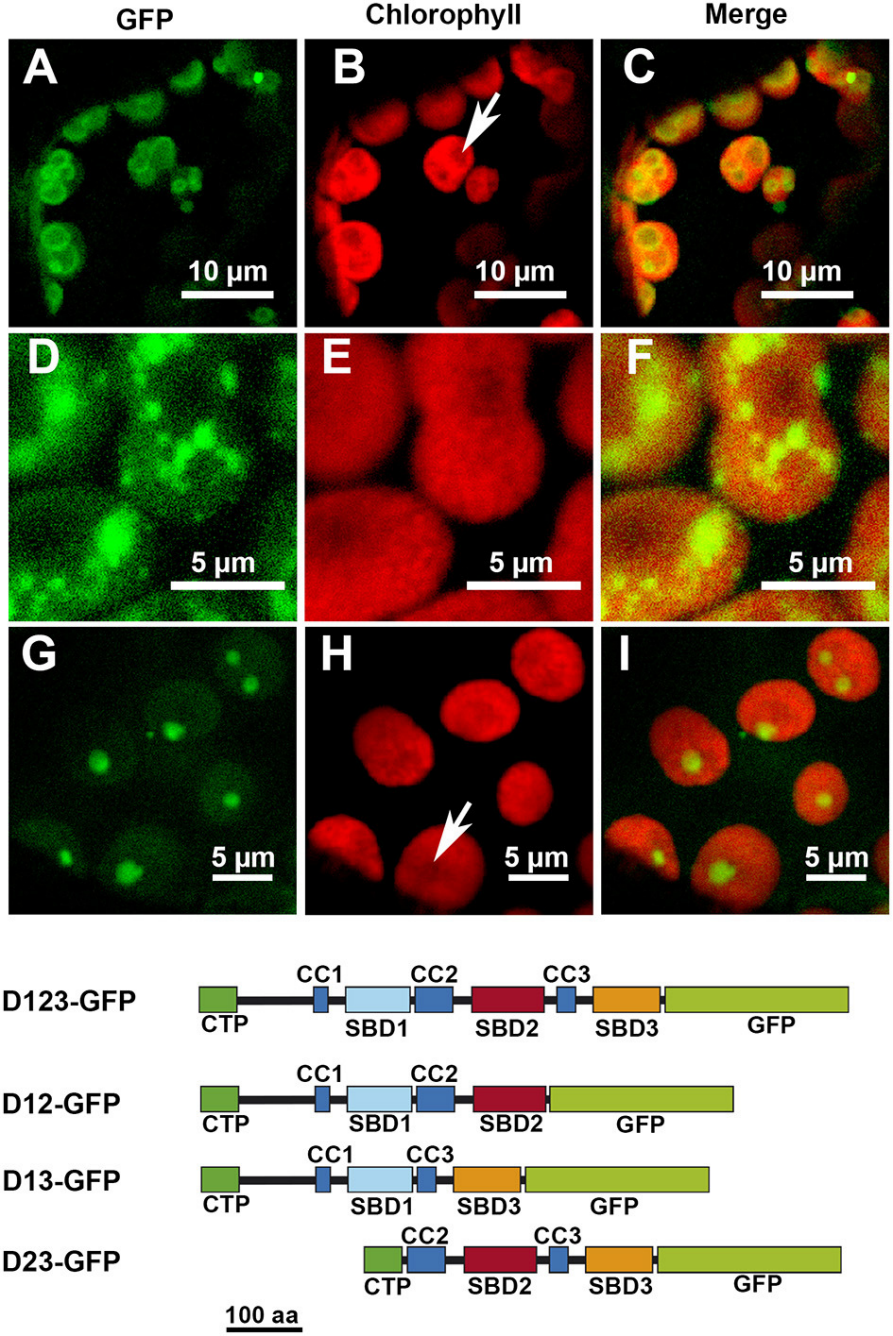
Localization of different truncated versions of AtSS3 fused to GFP in *N. benthamiana* chloroplasts. D12-GFP: **(A–C)**. D13-GFP: **(D–F)**. D23-GFP: **(G–I)**. GFP column: fluorescence of the GFP marker. Chlorophyll column: autofluorescence of the chlorophyll. Merge column: merged images of previous columns. Bottom: structures of the constructs fused to GFP. D12-GFP: N-terminal region of AtSS3 without D3 fused to GFP. D13-GFP: N-terminal region of AtSS3 without D2 fused to GFP. D23-GFP: N-terminal region of AtSS3 without D1 fused to GFP. The white arrows indicate the presence of a starch granule.

**Figure 4:**
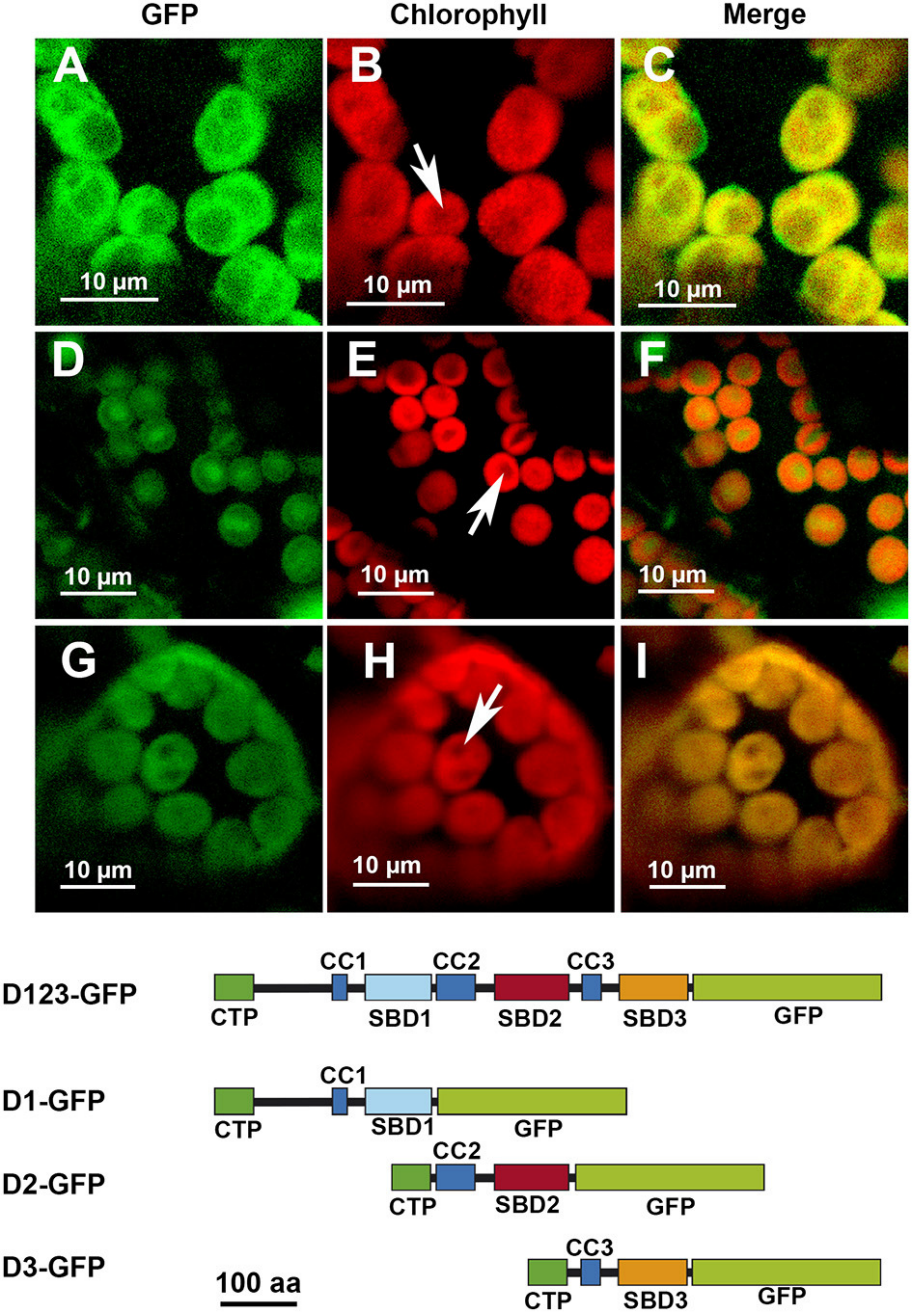
Localization of different truncated versions of AtSS3 fused to GFP in *N. benthamiana* chloroplasts. D1-GFP: **(A–C)**. D2-GFP: **(D–F)**. D3-GFP: **(G–I)**. GFP column: fluorescence of the GFP marker. Chlorophyll column: autofluorescence of the chlorophyll. Merge column: merged images of previous columns. Bottom: structures of the constructs fused to GFP. D1-GFP: N-terminal region of AtSS3 without D2 and D3 domains fused to GFP. D2-GFP: N-terminal region of AtSS3 without D1 and D2 domains fused to GFP. D2-GFP: N-terminal region of AtSS3 without D1 and D2 domains fused to GFP. The white arrows indicate the presence of a starch granule.

The removal of D3 did not seem to affect the localization pattern: the D12-GFP polypeptide displayed the same localization than D123-GFP (Figures 3A–C). The elimination of D2, however, modified the localization pattern, with the resulting D13-GFP polypeptide localizing preferentially in certain patches without apparent relation to any starch granules (Figures 3D–F). Finally, the elimination of D1 led to the resulting polypeptide, D23-GFP, localizing throughout the chloroplast but mainly in areas coincident with the starch granules (Figures 3G–I). D1 and D3 alone drove a similar pattern, with the resulting D1-GFP and D3-GFP polypeptides found all over the chloroplast (Figures 4A–C,G–I, respectively). D2-GFP showed a similar localization pattern to D23-GFP, with signals across the entire chloroplast, but especially concentrated in the regions coincident with the starch granules (Figures 4D–F).

### Presence of the AtSS3 Truncated Versions in the Starch

**Figure 5:**
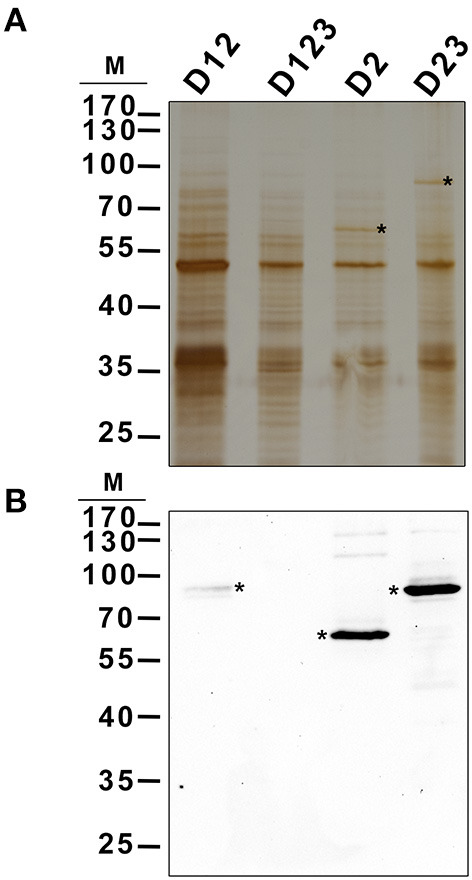
Localization of different AtSS3 truncated versions in starch of agroinfiltrated *N. benthamiana* leaves. The constructs D12-GFP, D123-GFP, D2-GFP and D23-GFP were transiently expressed in *N. benthamiana* leaves by agroinfiltration. Infiltrated leaves were harvested and starch was purified, washed and boiled to extract proteins localized in the polymer. 2 μg of protein were electrophoresed in SDS-PAGE and silver stained **(A)** or immunoblotted using anti-His5x antibody **(B)**. Asterisks indicate corresponding polypeptides. M, kDa marker.

The localization pattern of D23-GFP and D2-GFP seemed to indicate that these polypeptides co-localized with the starch granules. To determine whether these truncated polypeptides were bound to the polymer, their presence was sought on purified starch granules. Purified starch granules from *N. benthamiana* leaves agroinfiltrated with the constructs D12-, D123-, D2-, and D23-GFP were washed to eliminate proteins weakly bound to the polymer and then boiled to extract any proteins tightly bound to the polysaccharide. These proteins were analyzed by immunoblot using anti-His × 5 antibody (pEarleyGate 103 allows the C-terminus translational fusion of the fragment to GFP and to the 5 × His tail). This confirmed D2-GFP and D23-GFP to be tightly bound to the starch (Figure 5B). A faint band was also observed in the starch purified from D12-GFP infiltrated leaves, suggesting that its binding is weaker than that shown by D2-, and D23-GFP polypeptides. No signal was detected in the starch purified from leaves infiltrated with the D123-GFP polypeptide.

## Discussion

Most of the proteins involved in the starch metabolism are found in the soluble fraction of chloroplasts, although they must interact with the surface of the polymer, where its synthesis and degradation take place. Some of these proteins interact with the starch granule through SBDs (non-catalytic CBMs) whereas others, such as GBSS, require interaction with other polypeptides that contain a CBM. Indeed, the localization of GBSS in the starch granule requires an interaction with Protein Targeting To Starch 1, a polypeptide with a CC domain through which interacts with GBSS and a SBD domain belonging to the CBM48 family required for binding the protein to starch (Seung et al., 2015). Analysis of the interaction of glucan water dikinase and α-amylase 3 (both examples of starch metabolism proteins with SBDs belonging to the CBM45 family) with starch indicates that they bind with an affinity about two orders of magnitude lower than that of classical SBDs in microbial extracellular amylolytic enzymes. This suggests that low-affinity SBDs are a feature in plastidial starch metabolism, which allows binding to be reversible and facilitates the regulation of enzyme activities (Glaring et al., 2011).

SS3 contains three SBDs belonging to the CBM53 family (Palopoli et al., 2006). The functionality of the SBDs domains of *Arabidopsis* and *Ostreococcus tauri* SS3 have been comprehensively studied *in vitro* (Palopoli et al., 2006; Valdez et al., 2008; Wayllace et al., 2010; Barchiesi et al., 2015, 2018) and the results indicate that the N-terminal region shows an increased capacity to bind starch depending on the number of SBDs modules present. Moreover, the region containing SBD2 and 3 appear to makes the greatest contribution to binding (Valdez et al., 2008). The N-terminal SBDs also seem to have a regulatory role modulating the catalytic properties of SS3 (Valdez et al., 2008; Wayllace et al., 2010). We have previously shown that AtSS3 is localized to the area surrounding the starch granules, and that there is no strong interaction with the polysaccharide since the polypeptide is not found in the starch fraction after disrupting the chloroplasts (Gámez-Arjona et al., 2014). In the present work we show that the N-terminal region of AtSS3, which contains three SBDs, is sufficient to determine the same localization pattern shown by the full-length protein (Figure 1). In agreement with results of Valdez et al. (2008), SBD2 binds tightly to the starch granule, and the constructs D2-GFP and D23-GFP are detected in the purified starch granules of infiltrated *Nicotiana* leaves (Figure 5), likely because the tight binding of these polypeptides to the starch granule determine that they become trapped and buried in the growing polymer. The presence of D1 appears to weaken this interaction with the polymer, allowing the construct D12-GFP to show a localization pattern similar to that of the complete AtSS3. D1 seems to be more efficient than D3 at weakening the interaction of D2 with the starch granule (see the different localization pattern of D12-GFP and D23-GFP in Figure 3), but the presence of D12-GFP in the purified starch and the absence of D123-GFP (Figure 5) indicates that D3 is also necessary to prevent excessive binding. SBDs 1 and 3 seem unable to drive the localization of the polypeptide to the starch granule (see Figures 3D–F, 4A–C,G–I), supporting the idea that SBD2 is necessary for this to occur. Nevertheless, each domain contains a SBD and also a CC site, and it cannot be ruled out that some of the effect observed for the different construct are the consequence of the presence or elimination of these CC sites. It has been shown that SS3 form multiprotein complexes with other proteins of the starch metabolism in developing maize endosperm, and these interactions likely take place *via* the CC sites of the enzyme (Hennen-Bierwagen et al., 2008). Thus, the weakening effect of the SBD2-mediated interaction with starch observed for the D1 and D3 modules may be the consequence of interactions with other proteins exerted through their CC domains.

In summary, the *in planta* localization pattern of AtSS3 would appear to be the result of a balance between the different activities of the domains present in the N-terminal region of the enzyme.

## Data Availability Statement

The raw data supporting the conclusions of this article will be made available by the authors, without undue reservation.

## Author Contributions

FG-A conducted the experimental work. ÁM designed the work and wrote the manuscript. All authors contributed to the article and approved the submitted version.

## Funding

This work was funded by Grants BIO2012-35403 and PGC2018-096851-B-C22 from the Spanish Ministry of Science and Innovation (MICINN) and the European Fund for Regional Development.

## Conflict of Interest

The authors declare that the research was conducted in the absence of any commercial or financial relationships that could be construed as a potential conflict of interest.

## Publisher's Note

All claims expressed in this article are solely those of the authors and do not necessarily represent those of their affiliated organizations, or those of the publisher, the editors and the reviewers. Any product that may be evaluated in this article, or claim that may be made by its manufacturer, is not guaranteed or endorsed by the publisher.

## References

[B1] Abel G. J. W. Springer F. Willmitzer L. Kossmann J. (1996). Cloning and functional analysis of a cDNA encoding a novel 139 kDa starch synthase from potato (*Solanum tuberosum* L.). Plant J. 10, 981–991. 10.1046/j.1365-313X.1996.10060981.x 9011082

[B2] Abt M. R. Pfister B. Sharma M. Eicke S. Bürgy L. Neale I.. (2020). STARCH SYNTHASE5, a noncanonical starch synthase-like protein, promotes starch granule initiation in arabidopsis[OPEN]. Plant Cell 32, 2543–2565. 10.1105/tpc.19.00946 32471861 PMC7401018

[B3] Ball S. G. Morell M. K. (2003). From bacterial glycogen to starch: understanding the biogenesis of the plant starch granule. Annu. Rev. Plant Biol. 54, 207–233. 10.1146/annurev.arplant.54.031902.134927 14502990

[B4] Barchiesi J. Hedin N. Gomez-Casati D. F. Ballicora M. A. Busi M. V. (2015). Functional demonstrations of starch binding domains present in *Ostreococcus tauri* starch synthases isoforms. BMC Res. Notes 8:613. 10.1186/s13104-015-1598-6 26510916 PMC4625611

[B5] Barchiesi J. Velazquez M. B. Palopoli N. Iglesias A. A. Gomez-Casati D. F. Ballicora M. A.. (2018). Starch synthesis in *Ostreococcus tauri*: the starch-binding domains of starch synthase III-B are essential for catalytic activity. Front. Plant Sci. 9:e01541. 10.3389/fpls.2018.01541 30410499 PMC6210743

[B6] Buléon A. Colonna P. Planchot V. Ball S. (1998). Starch granules: structure and biosynthesis. Int. J. Biol. Macromol. 23, 85–112. 10.1016/S0141-8130(98)00040-3 9730163

[B7] Coutinho P. M. Deleury E. Davies G. J. Henrissat B. (2003). An evolving hierarchical family classification for glycosyltransferases. J. Mol. Biol. 328, 307–317. 10.1016/S0022-2836(03)00307-3 12691742

[B8] Denyer K. Barber L. M. Burton R. Hedley C. L. Hylton C. M. Johnson S.. (1995). The isolation and characterization of novel low-amylose mutants of Pisum sativum L. Plant Cell Environ. 18, 1019–1026. 10.1111/j.1365-3040.1995.tb00612.x

[B9] Earley K. W. Haag J. R. Pontes O. Opper K. Juehne T. Song K.. (2006). Gateway-compatible vectors for plant functional genomics and proteomics. Plant J. 45, 616–629. 10.1111/j.1365-313X.2005.02617.x 16441352

[B10] Edwards A. Fulton D. C. Hylton C. M. Jobling S. A. Gidley M. Rossner U.. (1999). A combined reduction in activity of starch synthases II and III of potato has novel effects on the starch of tubers. Plant J. 17, 251–261. 10.1046/j.1365-313X.1999.00371.x

[B11] Edwards A. Marshall J. Sidebottom C. Visser R. G. Smith A. M. Martin C. (1995). Biochemical and molecular characterization of a novel starch synthase from potato tubers. Plant J. 8, 283–294. 10.1046/j.1365-313X.1995.08020283.x 7670507

[B12] Fontaine T. D'Hulst C. Maddelein M. L. Routier F. Pepin T. M. Decq A.. (1993). Toward an understanding of the biogenesis of the starch granule. Evidence that Chlamydomonas soluble starch synthase II controls the synthesis of intermediate size glucans of amylopectin. J. Biol. Chem. 268, 16223–16230. 10.1016/S0021-9258(19)85409-1 8344907

[B13] Gámez-Arjona F. M. Raynaud S. Ragel P. Mérida Á. (2014). Starch synthase 4 is located in the thylakoid membrane and interacts with plastoglobule-associated proteins in Arabidopsis. Plant J. 80, 305–316. 10.1111/tpj.12633 25088399

[B14] Glaring M. A. Baumann M. J. Hachem M. A. Nakai H. Nakai N. Santelia D.. (2011). Starch-binding domains in the CBM45 family - low-affinity domains from glucan, water dikinase and α-amylase involved in plastidial starch metabolism. FEBS J. 278, 1175–1185. 10.1111/j.1742-4658.2011.08043.x 21294843

[B15] Hennen-Bierwagen T. A. Lin Q. Grimaud F. Planchot V. Keeling P. L. James M. G.. (2009). Proteins from multiple metabolic pathways associate with starch biosynthetic enzymes in high molecular weight complexes: a model for regulation of carbon allocation in maize amyloplasts. Plant Physiol. 149, 1541–1559. 10.1104/pp.109.135293 19168640 PMC2649383

[B16] Hennen-Bierwagen T. A. Liu F. Marsh R. S. Kim S. Gan Q. Tetlow I. J.. (2008). Starch biosynthetic enzymes from developing maize endosperm associate in multisubunit complexes. Plant Physiol. 146, 1892–1908. 10.1104/pp.108.116285 18281416 PMC2287357

[B17] Jeon J.-S. Ryoo N. Hahn T. R. Walia H. Nakamura Y. (2010). Starch biosynthesis in cereal endosperm. Plant Physiol. Biochem. 48, 383–392. 10.1016/j.plaphy.2010.03.006 20400324

[B18] Kuipers A. G. J. Jacobsen E. Visser R. G. F. (1994). Formation and deposition of amylose in the potato tuber starch granule are affected by the reduction of granule-bound starch synthase gene expression. Plant Cell 6, 43–52. 10.2307/3869673 12244219 PMC160414

[B19] Laemmli U. K. (1970). Cleavage of structural proteins during the assembly of the head of bacteriophage T4. Nature 227, 680–685. 10.1038/227680a0 5432063

[B20] Lowry O. H. Rosebrough N. J. Farr A. L. Randall R. J. (1951). Protein measurement with the Folin phenol reagent. J. Biol. Chem. 193, 265–275. 10.1016/S0021-9258(19)52451-6 14907713

[B21] Nakamura T. Yamamori M. Hirano H. Hidaka S. Nagamine T. (1995). Production of waxy (amylose-free) wheats. Mol. Gen. Genet. 1, 253–259. 10.1007/BF02191591 7565586

[B22] Palopoli N. Busi M. V. Fornasari M. S. Gomez-Casati D. Ugalde R. Parisi G. (2006). Starch-synthase III family encodes a tandem of three starch-binding domains. Proteins-Struct. Funct. Bioinforma. 65, 27–31. 10.1002/prot.21007 16862594

[B23] Roldán I. Wattebled F. Mercedes Lucas M. Delvallé D. Planchot V. Jiménez S.. (2007). The phenotype of soluble starch synthase IV defective mutants of *Arabidopsis thaliana* suggests a novel function of elongation enzymes in the control of starch granule formation. Plant J. 49, 492–504. 10.1111/j.1365-313X.2006.02968.x 17217470

[B24] Rose A. Meier I. (2004). Scaffolds, levers, rods and springs: diverse cellular functions of long coiled-coil proteins. Cell. Mol. Life Sci. 61, 1996–2009. 10.1007/s00018-004-4039-6 15316650 PMC11138566

[B25] Seung D. Soyk S. Coiro M. Maier B. A. Eicke S. Zeeman S. C. (2015). Protein targeting to starch is required for localising granule-bound starch synthase to starch granules and for normal amylose synthesis in Arabidopsis. PLoS Biol. 13:e1002080. 10.1371/journal.pbio.1002080 25710501 PMC4339375

[B26] Szydlowski N. Ragel P. Hennen-Bierwagen T. A. Planchot V. Myers A. M. Mérida A.. (2011). Integrated functions among multiple starch synthases determine both amylopectin chain length and branch linkage location in Arabidopsis leaf starch. J. Exp. Bot. 62, 4547–4559. 10.1093/jxb/err172 21624979

[B27] Szydlowski N. Ragel P. Raynaud S. Lucas M. M. Roldan I. Montero M.. (2009). Starch granule initiation in Arabidopsis requires the presence of either class IV or class III starch synthases. Plant Cell 21, 2443–2457. 10.1105/tpc.109.066522 19666739 PMC2751949

[B28] Valdez H. A. Busi M. V. Wayllace N. Z. Parisi G. Ugalde R. A. Gomez-Casati D. F. (2008). Role of the N-Terminal Starch-Binding Domains in the Kinetic Properties of Starch Synthase III from *Arabidopsis thaliana*. Biochemistry 47, 3026–3032. 10.1021/bi702418h 18260645

[B29] Wayllace N. Z. Valdez H. A. Ugalde R. A. Busi M. V. Gomez-Casati D. F. (2010). The starch-binding capacity of the noncatalytic SBD2 region and the interaction between the N- and C-terminal domains are involved in the modulation of the activity of starch synthase III from *Arabidopsis thaliana*: enzymes and catalysis. FEBS J. 277, 428–440. 10.1111/j.1742-4658.2009.07495.x 19968859

[B30] Wood D. W. Setubal J. C. Kaul R. Monks D. E. Kitajima J. P. Okura V. K.. (2001). The genome of the natural geneticengineer *Agrobacterium tumefaciens* C58. Science 294, 2317–2323. 10.1126/science.1066804 11743193

[B31] Zhang X. Myers A. M. James M. G. (2005). Mutations affecting starch synthase III in Arabidopsis alter leaf starch structure and increase the rate of starch synthesis. Plant Physiol. 138, 663–674. 10.1104/pp.105.060319 15908598 PMC1150387

[B32] Zimmermann L. Stephens A. Nam S. Z. Rau D. Kübler J. Lozajic M.. (2018). A completely reimplemented MPI bioinformatics toolkit with a new HHpred server at its core. J. Mol. Biol. 430, 2237–2243. 10.1016/j.jmb.2017.12.007 29258817

