# Supplementary material for: Interplay Between the N-Terminal Domains of Arabidopsis Starch Synthase 3 Determines the Interaction of the Enzyme With the Starch Granule
Source: Front Plant Sci. 2021 Sep 23;12:704161. doi: 10.3389/fpls.2021.704161 (PMC8494965; doi:10.3389/fpls.2021.704161)
Supplement: Supplementary file 1 [file Table_1.DOCX]

**Supplementary Table 1. Primers used to clone the different truncated versions of AtSS3**

| **FRAGMENT** | **OLIGONUCLEOTIDES** |
| --- | --- |
| D123 | SS3_GATEWAY_F GGGGACAAGTTTGTACAAAAAAGCAGGCTTCATGGAAGTGTGTTGGCAGATA  SS3_Nterm_GATEWAY_R GGGGACCACTTTGTACAAGAAAGCTGGGTCGTGCAATGGTGGTTCCTTTGA |
| D1 | SS3_GATEWAY_F GGGGACAAGTTTGTACAAAAAAGCAGGCTTCATGGAAGTGTGTTGGCAGATA  SS3_BD1_R GGG GAC CAC TTT GTA CAA GAA AGC TGG GTC AAC TTT ATC CAT CCC |
| D2 | SS3_GATEWAY_F GGGGACAAGTTTGTACAAAAAAGCAGGCTTCATGGAAGTGTGTTGGCAGATA  SS3_CTP_SBD2_R ATCCATCCCACCTTTATTTGATTTGTATTG  SS3_CTP_SBD2_F CAATACAAATCAAATAAAGGTGGGATGGAT  SS3_BD12_R GGGGACCACTTTGTACAAGAAAGCTGGGTCTTCTTCAGGAAGTTTTTGAGG |
| D3 | SS3_GATEWAY_F GGGGACAAGTTTGTACAAAAAAGCAGGCTTCATGGAAGTGTGTTGGCAGATA  SS3_CTP-SBD3_R AGG AAGTTTTTGAGGATTTGATTTGTATTG  SS3_CTP-SBD3_F CAATACAAATCAAATCCTCAAAAACTTCCT  SS3_Nterm_GATEWAY_R GGGGACCACTTTGTACAAGAAAGCTGGGTCGTGCAATGGTGGTTCCTTTGA |
| D12 | SS3_GATEWAY_F GGGGACAAGTTTGTACAAAAAAGCAGGCTTCATGGAAGTGTGTTGGCAGATA  SS3_BD12_R GGGGACCACTTTGTACAAGAAAGCTGGGTCTTCTTCAGGAAGTTTTTGAGG |
| D13 | SS3_GATEWAY_F GGGGACAAGTTTGTACAAAAAAGCAGGCTTCATGGAAGTGTGTTGGCAGATA  SS3_SBD13_R AGGAAGTTTTTGAGG**TATCTCTACACAAAA**  SS3_SBD13_F **TTTTGTGTAGAGATA**CCTCAAAAACTTCCT  SS3_Nterm_GATEWAY_R GGGGACCACTTTGTACAAGAAAGCTGGGTCGTGCAATGGTGGTTCCTTTGA |
| D23 | SS3_GATEWAY_F GGGGACAAGTTTGTACAAAAAAGCAGGCTTCATGGAAGTGTGTTGGCAGATA  SS3_CTP_SBD2_R ATCCATCCCACCTTTATTTGATTTGTATTG  SS3_CTP_SBD2_F CAATACAAATCAAATAAAGGTGGGATGGAT  SS3_Nterm_GATEWAY_R GGGGACCACTTTGTACAAGAAAGCTGGGTCGTGCAATGGTGGTTCCTTTGA |

In red the attb sequences of the GATEWAY system. Underlined the sequence corresponding to the chloroplast transit peptide. In bold and underlined the sequence corresponding to D1
